# Mealtime irregularity is associated with higher serum growth differentiation factor-15 levels in older adults: the explanatory role of dietary quality

**DOI:** 10.1007/s41999-026-01524-9

**Published:** 2026-06-16

**Authors:** Kaori Kinoshita, Yosuke Osuka, Georg von Fingerhut, Kazuhiro Yoshiura, Noriko Hori, Shosuke Satake, Shigenobu Shibata, Hidenori Arai

**Affiliations:** 1 Department of Frailty Research, Center for Gerontology and Social Science, Research Institute, National Center for Geriatrics and Gerontology, 7-430, Morioka-Cho, Obu, Aichi 474-8511 Japan; 2 Department of Geriatric Medicine, Hospital, National Center for Geriatrics and Gerontology, Obu, Aichi Japan; 3 Department of Public Health and Health Policy, Graduate School of Biomedical and Health Sciences, Hiroshima University, Hiroshima, Japan; 4 National Center for Geriatrics and Gerontology, Obu, Aichi Japan

**Keywords:** Mealtime irregularity, Biological aging, Dietary quality, Older adults, Mediation analysis, Chronononutrition

## Abstract

**Purpose:**

This study examined the association between mealtime irregularity and Growth Differentiation Factor-15 (GDF-15)—a potential biomarker of biological aging—and evaluated whether this association could be explained by poor dietary quality among community-dwelling older adults.

**Methods:**

This cross-sectional study analyzed data from 378 disability-free adults aged ≥ 65 years from the Higashiura Study. Eating habits (regular vs. irregular) were assessed during the interviews. Dietary quality was evaluated using nutrient density scores from the Nutrient-Rich Food Index, calculated from 3-day dietary records. The association among mealtime irregularity, dietary quality, and serum GDF-15 levels was examined using mediation analysis after adjusting for age, sex, comorbidities, sleep duration, physical activity, smoking status, employment status, and total energy intake.

**Results:**

Participants with irregular mealtimes were significantly younger than those with regular mealtimes (mean ± standard deviation: 73.3 ± 4.6 vs. 75.1 ± 5.6 years, *p* = 0.014) and had higher serum GDF-15 levels (least-squares means, 95% confidence interval [CI]: 1161.4, 1063.4–1270.2 vs. 1028.0, 988.4–1068.9 pg/mL, *p* = 0.009). In the mediation analysis, lower nutrient density partially explained the association between mealtime irregularity and higher GDF-15 levels (β, 95% CI: total effect: 0.053, 0.0078–0.0977; direct effect: 0.046, 0.0004–0.0898; indirect effect: 0.008, 0.0001–0.0205; mediation proportion: 14.5%).

**Conclusion:**

Although participants with irregular mealtimes were significantly younger than those with regular mealtimes, they showed higher levels of biological aging markers. Dietary quality partially accounted for this association. These findings suggest that dietary quality may be an important consideration for older adults with irregular mealtimes.

## Key summary points

**Aim:**

To examine the association between meal irregularity and serum GDF-15 levels—a potential biomarker of biological aging—and to determine whether dietary quality mediates this association in community-dwelling older adults.

**Findings:**

Participants with irregular mealtimes were significantly younger than those with regular mealtimes, yet they exhibited higher serum GDF-15 levels and lower dietary quality. Mediation analysis indicated that poor dietary quality partially accounted for the association between mealtime irregularity and elevated GDF-15 levels.

**Message:**

Improving dietary quality may be an important consideration for older adults with irregular mealtimes.

## Introduction

Healthy aging is an urgent public health priority [1]. Diet is a key modifiable factor for maintaining the health of older adults. Recently, an increasing number of studies have examined the role of circadian rhythms in the aging process [2], highlighting the importance of dietary strategies that consider biological rhythms—not only *what* to eat, but also *when* to eat.

Irregular mealtimes disrupt biological rhythms and are associated with metabolic diseases [3, 4] that accelerate key aging pathways, including impaired nutrient sensing, telomere shortening, epigenetic alterations, and mitochondrial dysfunction [5–9]; i.e., irregular meal timing may accelerate biological aging. However, irregular mealtimes frequently arise from unavoidable circumstances such as shift work. As the population ages, the number of older adults in the workforce increases. Therefore, strategies to slow the progression of biological aging may become increasingly important in older individuals with irregular mealtimes. Among these strategies, improving dietary quality may be particularly relevant.

It has become increasingly evident that chronological and biological age do not necessarily progress in parallel, underscoring the importance of lifestyle modifications in slowing biological aging. Growth Differentiation Factor-15 (GDF-15) is a biomarker that reflects biological aging [10, 11] and is highly sensitive to mitochondrial dysfunction [12, 13]. Previous studies have shown that GDF-15 levels increase before the clinical manifestations of aging, such as declines in physical function, become apparent [10, 11, 14]. Therefore, GDF-15 is considered a promising marker of biological aging. Although no prior study has directly examined the relationship between irregular mealtimes and biomarkers of aging, a recent observational study reported that meal timing patterns in older adults were associated with mortality risk [15]. Previous studies have reported inverse associations between dietary quality and GDF-15 levels [16–18], and mealtime irregularity has been linked to poor dietary quality [4, 19–21]. Diet quality also plays a key role in the development of metabolic diseases [22, 23].

Overall, these findings indicate that mealtime irregularity may be associated with biological aging, and that poor dietary quality could partially explain this association. Accordingly, based on prior conceptual considerations, we hypothesized that irregular mealtimes may accelerate biological aging through lower dietary quality. However, no previous study has directly examined this hypothesis-driven relationship. Clarifying this association would provide useful insights for developing dietary strategies to slow biological aging among older adults with irregular mealtimes. Moreover, it is also important to identify which aspects of dietary intake should be emphasized for older adults whose mealtimes are irregular and unlikely to change. Therefore, this study aimed to examine the association between mealtime irregularity and GDF-15 levels and to explore the extent to which dietary quality—and its food components—accounts for this relationship in community-dwelling older adults.

## Methods

### Study design and participants

This cross-sectional study was conducted among community-dwelling adults aged ≥ 65 years without disabilities in Higashiura Town, Aichi, Japan (Higashiura Study). Higashiura Town, located in central Japan, covers an area of 31.14 km^2^ and has a population of approximately 50,000 residents living in 21,000 households [24].

**Fig. 1:**
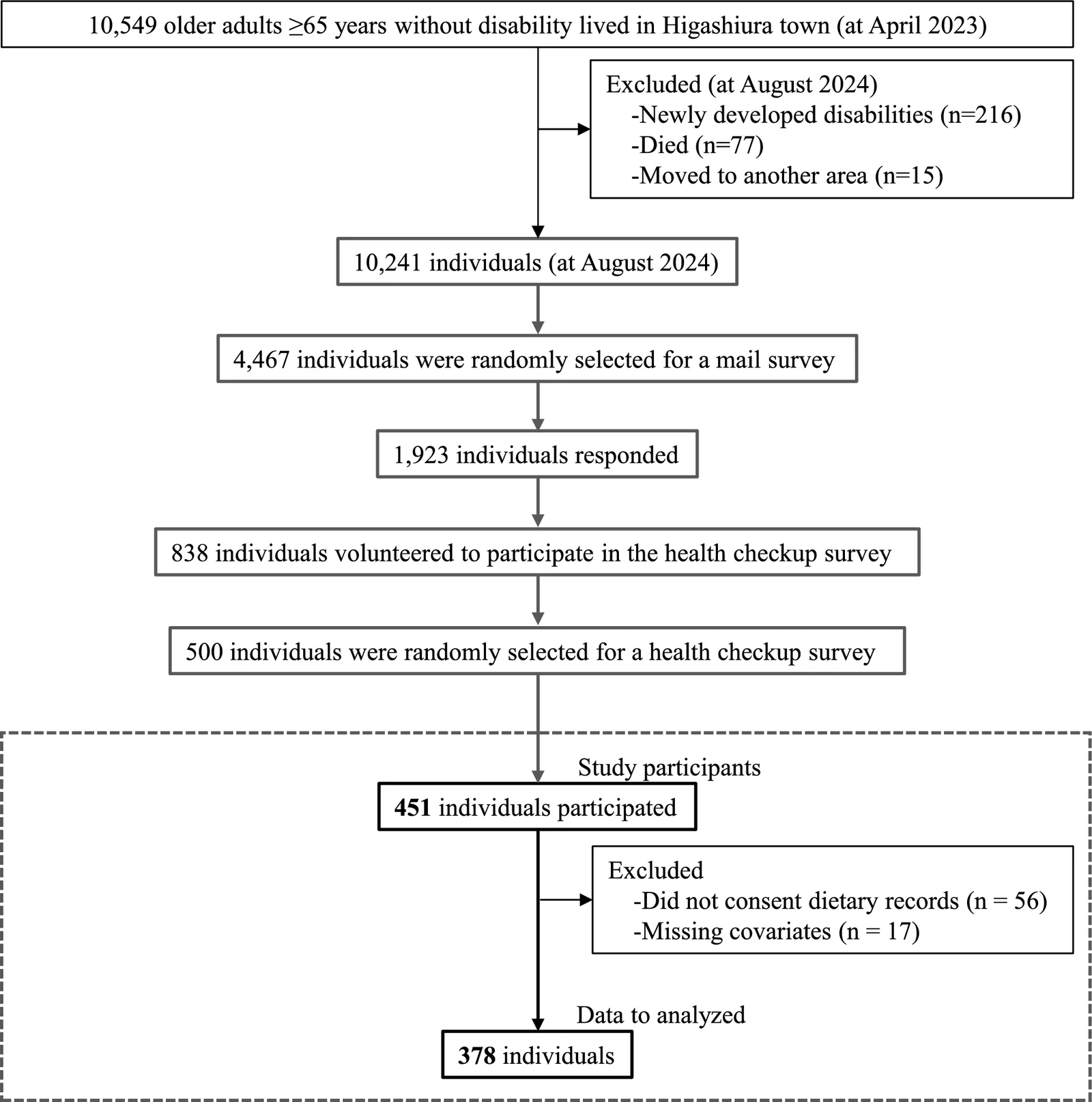
Participants selection flowchart. The study comprised two phases: a mail survey and a health checkup survey. First, we obtained from the local government a list of 10,549 residents aged ≥ 65 years without disabilities as of April 1, 2023. From this list, we excluded 216 individuals with newly developed disabilities, 77 who died, and 15 who moved out of the area by August 2024. Of the remaining eligible population, 4,467 individuals were randomly selected for mail surveys. The survey collected information on current illnesses, living conditions, and related factors, with 1,923 individuals responding. Among these respondents, 838 volunteered to participate in the subsequent health checkup survey, of which 500 were randomly selected. Of these, 49 individuals could not attend the health checkup for reasons, including poor health on the survey day. The final sample comprised 451 older adults

The study comprised two phases: a mail survey and a health checkup survey. First, we obtained a list of 10,549 residents aged ≥ 65 years without disabilities as of April 1, 2023, from the local government. From this list, we excluded 216 individuals with newly developed disabilities, 77 who died, and 15 who moved out of the area by August 1, 2024. Of the remaining eligible population, 4,467 individuals were randomly selected for mail surveys. The survey collected information on current illnesses, living conditions, and related factors, with 1,923 individuals responding. Among these respondents, 838 volunteered to participate in the subsequent health checkup survey, of which 500 were randomly selected. Of these, 49 individuals could not attend the health checkup for reasons, including poor health on the survey day. A total of 451 older individuals were included in this study (Fig. 1).

### Ethics statement

This study was conducted in accordance with the Declaration of Helsinki, and all procedures were approved by the Ethics Committee for Human Research of the National Center for Geriatrics and Gerontology, Japan (No. 1952). All participants provided written informed consent.

### Outcome variable: measurements of serum GDF-15 levels

Blood samples were collected onsite during the health checkup via standard venipuncture of the antecubital vein. Immediately after collection, the samples were centrifuged, and the serum was stored at − 80 °C until analysis. Serum GDF-15 concentrations (pg/mL) were measured using a commercially available enzyme-linked immunosorbent assay kit (Human GDF-15 Quantikine Quick ELISA; R&D Systems, Minneapolis, MN, USA).

### Explanatory variable: assessment of mealtime irregularity

In this study, mealtime irregularity was defined as meals consumed at varying times from one day to the next. It was assessed using an interviewer-administered questionnaire conducted at the health checkup site. Research staff asked participants about their usual mealtime habits over the past month. Specifically, for each meal—breakfast, lunch, and dinner—participants were asked: “Do you think you usually eat this meal at the same time every day?” Response options were: “strongly agree,” “agree,” “neither agree nor disagree,” “disagree,” and “strongly disagree.” For each meal, participants who answered “strongly agree” or “agree” were classified as having a regular mealtime, whereas all other responses were classified as irregular. Participants who reported irregular mealtimes for at least one of the three meals (i.e., breakfast, lunch, or dinner) were assigned to the irregular mealtime group.

Because no unified standard exists for assessing mealtime irregularity [25], we designed a study-specific questionnaire informed by prior research using subjective Likert-type measures [26] and by expert consultation. Importantly, previous work has shown that subjective reports of meal timing deviate from objectively recorded timing by less than 10% [27].

### Mediator variable: assessment of dietary quality

On the day of the health checkup, the participants were instructed to complete a 3-day dietary record (3DR) at home. Those who agreed to participate recorded their food intake for 2 weekdays and 1 weekend day within 2 weeks of returning home.

The participants photographed their meals before and after eating and submitted these images along with their dietary records. A trained dietitian, using a standardized coding manual, coded all food items based on the written records and accompanying photographs. When leftover food was visible in post-meal photographs, the dietitian verified that it was accurately reflected in the dietary records. For example, if a participant recorded consuming a full portion of soup but the post-meal photograph showed that part of it was left uneaten, the dietitian corrected the estimated intake accordingly. After these corrections, dietitians calculated the average dietary intake over 3 days using the Standard Tables of Food Composition in Japan [28].

Diet quality was assessed using the Nutrient-Rich Food Index (NRF) [29–31]. The NRF is a composite indicator of dietary nutrient density based on qualifying nutrients (NR) and disqualifying nutrients (LIM) [29–31]. In this study, we used the NRF9.2 for total diets, which incorporates nine NR nutrients (protein, dietary fiber, vitamin A, vitamin C, vitamin D, calcium, iron, potassium, and magnesium) and two LIM nutrients (saturated fat and sodium). In the method proposed in the previous studies [30, 31], added sugars are included as LIM components. However, the Japanese Standard Tables of Food Composition do not provide values for added sugars [28]. Therefore, added sugars were not included in the NRF calculation.

Following the approach of Murakami et al. [29], nutrient intake was standardized according to each participant's estimated energy requirement. The NRF9.2 was calculated as the sum of percentages of reference daily values for nine NR nutrients, minus the sum of percentages of maximum reference daily values for two LIM nutrients. For NR nutrients, percentages were truncated at 100% to prevent excessive intake of one nutrient from compensating for inadequacies in others. For LIM nutrients, only the proportion exceeding the recommended limit was counted. A higher NRF9.2 indicates better diet quality. The maximum possible score of 900 represents a diet that meets the recommended intake for all nine NR nutrients while remaining within the upper limits for the two LIM nutrients.

### Other measurements

Height, weight, blood pressure, heart rate, and waist circumference were measured during the health checkups. Blood tests were used to assess hemoglobin, blood urea nitrogen, creatinine, estimated glomerular filtration rate, triglycerides, low-density lipoprotein cholesterol, aspartate aminotransferase, alanine aminotransferase, γ-glutamyl transpeptidase, hemoglobin A1c, and high-sensitivity C-reactive protein. Comorbidities (hypertension, stroke, heart disease, diabetes, kidney disease, digestive disease, respiratory disease, and cancer) were confirmed using a self-administered questionnaire and verified in person by a nurse. Additionally, smoking status (current smoker or nonsmoker), employment status (employed or unemployed), self-reported sleep duration (hours/day), and regular physical activity (dichotomized into regular activity—light exercise, gymnastics, or sports at least once per week—and none) were assessed.

### Statistical analysis

Participant characteristics were compared between the irregular and regular mealtime groups using one-way analysis of variance for continuous variables and the chi-square test (or Fisher’s exact test when the expected frequency was < 5) for categorical variables.

Analysis of covariance was applied to compare GDF-15 levels and NRF9.2 scores between the two groups. GDF-15 levels were log10-transformed, and the covariates included sex, age, number of comorbidities, sleep duration, physical activity, smoking status, employment status, and energy intake.

For the main analysis, a mediation analysis with 1,000 bootstrap resamples was conducted, with mealtime irregularity, NRF9.2, and GDF-15 level as the independent, mediating, and dependent variables, respectively. The covariates listed above were included. To facilitate interpretation, NRF9.2 scores were scaled in 100-point increments, and GDF-15 levels were log10-transformed. Mediation analysis was performed using the “mediation” package in R. Given the cross-sectional design, temporal ordering among irregular mealtimes, dietary quality, and GDF-15 levels cannot be established, and the analysis does not aim to infer causality. Accordingly, the mediation analysis should be interpreted as a statistical decomposition of the observed associations rather than as causal mediation.

To evaluate the relative contribution of each food group to the variance in NRF9.2, we applied the Lindeman–Merenda–Gold (LMG) method. The LMG method estimates the relative importance of predictors in multiple regression by partitioning R^2^ while accounting for multicollinearity. This method is widely used to evaluate relative importance in multicollinear datasets, such as those involving dietary intake [32, 33]. LMG analysis was conducted using the “relaimpo” package in R, and the covariates used in the mediation analysis were also included. Differences in food group intake between the irregular and regular mealtime groups were also examined using analysis of covariance with the same covariates.

All statistical analyses were performed using IBM SPSS Statistics for Windows, version 28.0 (IBM Corp., Armonk, NY, USA) and R software (version 4.5.0; R Foundation for Statistical Computing, Vienna, Austria). Statistical significance was defined as a two-sided *p*-value of < 0.05.

## Results

Of the 451 participants enrolled in this study, data from 378 were included in the analysis after excluding 56 who did not consent to 3DR and 17 with missing covariates.

Table 1 presents the characteristics of the study participants. The irregular mealtime group was significantly younger than the regular mealtime group (73.3 ± 4.6 vs. 75.1 ± 5.6 years, *p* = 0.014). The irregular mealtime group also had a higher prevalence of skipped meals, with breakfast being the most frequently skipped meal, followed by lunch and dinner. Mean arterial pressure, an indicator of peripheral arteriosclerosis, was significantly higher in the irregular mealtime group than in the regular mealtime group (109.5 ± 16.8 vs. 104.8 ± 13.4 mmHg, *p* = 0.014). Among males, waist circumference was significantly greater in the irregular mealtime group than in the regular mealtime group (90.0 ± 9.7 vs. 86.8 ± 10.1 cm, *p* = 0.041).

**Table 1:** Characteristics of the participants

	Irregular mealtimes (*n* = 63)	Regular mealtimes (*n* = 315)	*p*-value
Male sex, n (%)	30 (47.6)	158 (50.2)	0.409
Age, years	73.3 ± 4.6	75.1 ± 5.6	0.014
BMI, kg/m^2^	23.6 ± 3.6	22.9 ± 3.0	0.090
Current smoker, n (%)	5 (7.9)	21 (6.7)	0.443
Employment status, n (%)	23 (36.5)	89 (28.3)	0.114
Self-reported sleep duration, hours/day	6.8 ± 1.3	7.0 ± 1.1	0.255
Engages in regular physical activity, n (%)	11 (17.5)	50 (15.9)	0.439
Meal skipping, n (%)	15 (23.8)	9 (2.9)	< 0.001
Skip breakfast	7 (11.1)	7 (2.2)	< 0.001
Skip lunch	5 (7.9)	1 (0.3)	
Skip dinner	3 (4.8)	1 (0.3)	
Hypertension, n (%)	34 (54.0)	140 (44.4)	0.107
Stroke, n (%)	3 (4.8)	13 (4.1)	0.516
Heart disease, n (%)	8 (12.7)	41 (13.0)	0.568
Diabetes, n (%)	3 (4.8)	32 (10.2)	0.130
Kidney disease, n (%)	2 (3.2)	16 (5.1)	0.398
Digestive disease, n (%)	10 (15.9)	66 (21.0)	0.231
Respiratory disease, n (%)	10 (15.9)	23 (7.3)	0.031
Cancer, n (%)	9 (14.3)	35 (11.1)	0.298
Number of comorbidities	1 [0, 2]	1 [0, 2]	0.875
BUN, mg/dL	16.8 ± 4.5	17.4 ± 4.6	0.293
Cr, mg/dL	0.8 ± 0.3	0.8 ± 0.2	0.357
TG, mg/dL	128.2 ± 70.1	128.7 ± 72.6	0.960
AST, U/L	23.3 ± 4.8	23.7 ± 7.1	0.615
ALT, U/L	19.8 ± 8.6	20.4 ± 9.9	0.685
γ-GT, U/L	29.5 ± 27.1	29.9 ± 24.3	0.900
LDL-C, mg/dL	127.1 ± 31.0	126.2 ± 31.2	0.842
eGFR, mL/min/1.73m^2^	63.8 ± 13.4	65.0 ± 12.5	0.502
Hb, g/dL	14.2 ± 1.6	14.0 ± 1.4	0.438
HbA1c, %	5.7 ± 0.5	5.8 ± 0.6	0.171
hs-CRP, mg/dL	0.3 ± 1.7	0.3 ± 1.7	0.487
Waist circumference, cm			
Male	90.9 ± 9.7	86.8 ± 10.1	0.041
Female	84.5 ± 11.3	84.8 ± 9.7	0.867
SBP, mmHg	153.9 ± 25.5	149.2 ± 22.0	0.143
DBP, mmHg	87.3 ± 15.3	82.5 ± 12.2	0.006
HR, bpm	71.1 ± 11.5	70.5 ± 11.3	0.734
PP^*^, mmHg	66.5 ± 20.3	66.8 ± 19.1	0.923
MAP^†^, mmHg	109.5 ± 16.8	104.8 ± 13.4	0.014
Number of MAP ≧90 mmHg, n (%)	57 (90.5)	274 (87.0)	0.297

*ALT* alanine aminotransferase, *AST* aspartate aminotransferase, *BUN* blood urea nitrogen, *Cr* creatinine, *DBP* diastolic blood pressure, *eGFR* estimated glomerular filtration rate, *Hb* hemoglobin, *HbA1c* hemoglobin A1c, *HR* heart rate, *hs-CRP* high-sensitivity C-reactive protein, *MAP* mean arterial pressure, *PP* pulse pressure, *SBP* systolic blood pressure, *TG* triglyceride, *γ-GT* γ-glutamyl transpeptidase, *LDL-C* low-density lipoprotein cholesterol

Values are presented as mean ± standard deviation, median [interquartile range], or n (%)

^*^PP = SBP − DBP

^†^MAP = SBP + (2 × DBP)/3

After adjusting for covariates, the irregular mealtime group had significantly higher GDF-15 levels and lower NRF9.2 scores than the regular mealtime group. The least-squares means (LSM) and 95% confidence intervals (CIs) were as follows: GDF-15,1161.4 (1063.4–1270.2) vs. 1028.0 (988.4–1068.9) pg/mL, *p* = 0.009; NRF9.2, 798.3 (781.7–815.0) vs. 823.2 (815.9–830.6) points, *p* = 0.008.

**Fig. 2:**
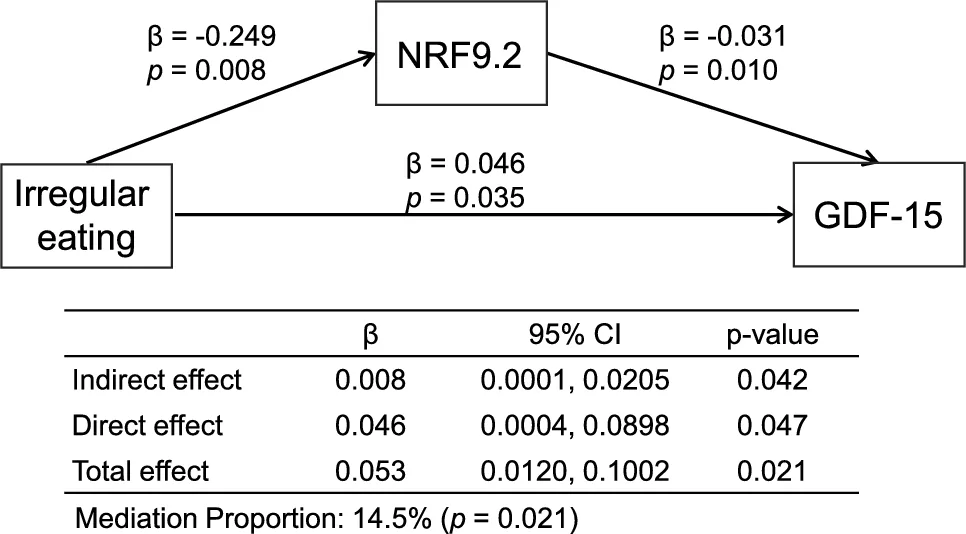
Mediation analysis results of irregular eating, dietary quality, and biological aging marker. Mediation analysis with 1,000 bootstrap resamples was conducted with irregular eating, NRF9.2, and GDF-15 level as the independent, mediating, and dependent variables, respectively. The covariates were sex, age, number of comorbidities, sleep duration, physical activity, smoking status, employment status, and energy intake. Irregular eating is significantly associated with higher GDF-15 levels through lower NRF9.2. GDF-15, Growth Differentiation Factor-15; NRF, Nutrient-Rich Food Index

Figure 2 shows the results of the mediation analysis. Irregular mealtimes were significantly associated with higher GDF-15 levels through lower NRF9.2 scores (indirect effect: β = 0.008, 95% CI: 0.0001–0.0205, *p* = 0.042; direct effect: β = 0.046, 95% CI: 0.0004–0.0898, *p* = 0.047; total effect: β = 0.053, 95% CI: 0.0120–0.1002, *p* = 0.021). The proportion mediated by NRF9.2 was 14.5% (*p* = 0.021).

**Fig. 3:**
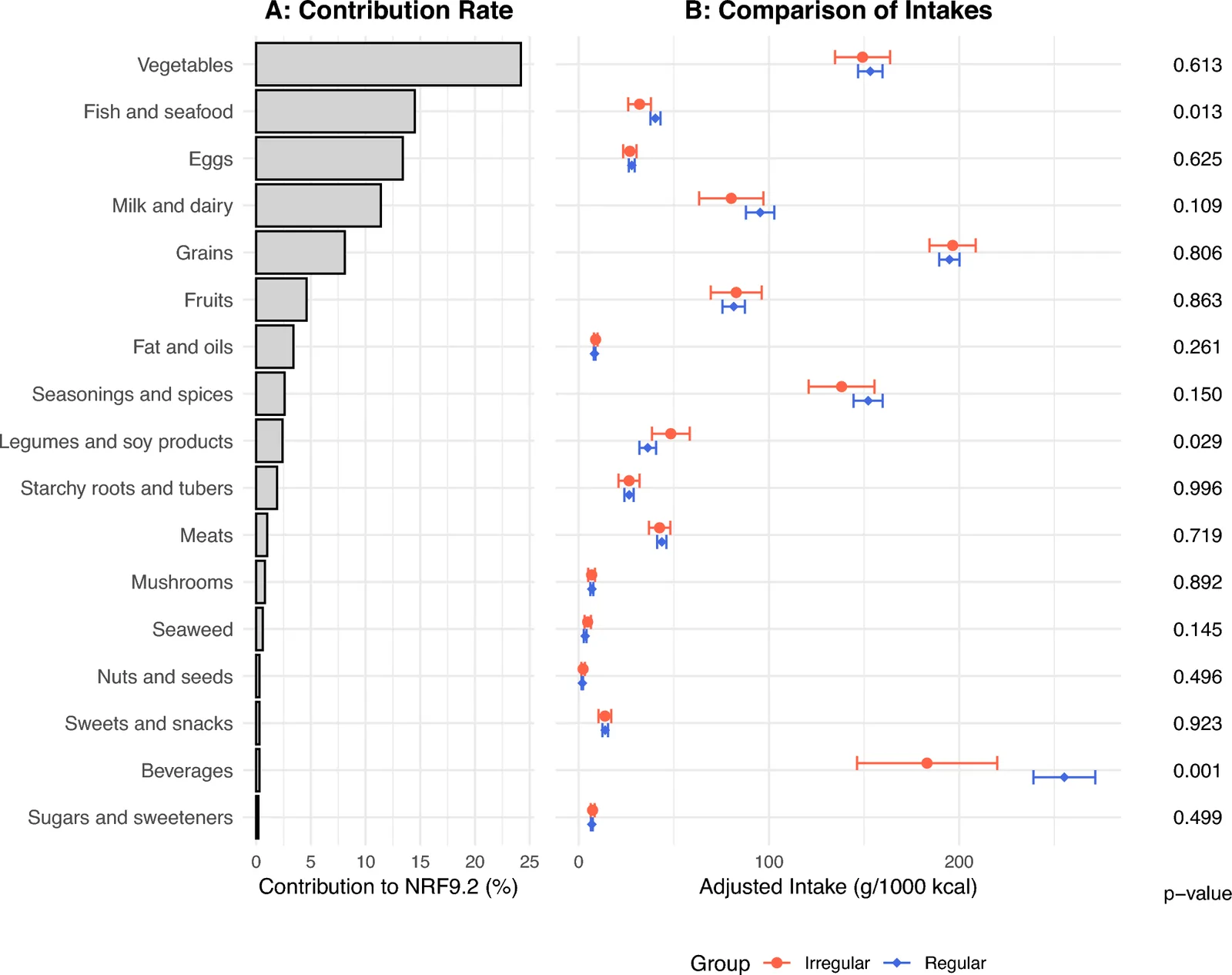
Integrated analysis of food group contributions to NRF9.2 and comparison of intakes between mealtime groups. **A** Contribution to NRF9.2 (%): Bar chart illustrating the relative contribution of each food group to the variance of NRF9.2. Contribution rates were calculated using the LMG method, adjusted for sex, age, number of comorbidities, sleep duration, physical activity, smoking status, employment status, and total energy intake. Food groups are ranked in descending order of relative importance. B Comparison of intakes: Forest plot showing the LSM intakes (g/1000 kcal) for the irregular mealtime group (red circles) and the regular mealtime group (blue diamonds). Points represent LSM, and error bars indicate 95% CIs. Values were estimated using an ANCOVA model adjusted for the same covariates used in the LMG analysis. *p*-value: The right column shows *p*-values for inter-group comparisons from the ANCOVA. Statistical significance was defined as *p* < 0.05. Note: Seasoning and spices included soy sauce, miso, vinegar, salt, sugar, cooking alcohol, various sauces, mayonnaise, curry and chili powders, herbs, and other spice mixtures commonly used to flavor foods. Beverages included tea, coffee, soft drinks, fruit juices, and alcoholic beverages as defined in the Japanese food classification. Sugars and sweeteners included table sugar, honey, syrups, and other sweetening agents as defined in the Japanese Food Classification. This category represents sugars consumed as food and excludes sugars added during the manufacture of processed foods or beverages. *ANCOVA* analysis of covariance, *CI* confidence interval, *LMG* Lindeman–Merenda–Gold, *LSM* least-squares means, *NRF9.2* Nutrient-Rich Food Index 9.2

The results of the LMG analysis examining the contribution of food groups to NRF9.2 are presented in Fig. 3. Vegetables made the largest contribution to NRF9.2 (24.2%), followed by fish and seafood (14.5%) and eggs (13.4%). The intake of fish and seafood was significantly lower in the irregular mealtime group than in the regular mealtime group (LSM and 95% CI: 32.0 [30.0–38.0] vs. 40.3 [37.7–43.0] g/1000 kcal, *p* = 0.013).

## Discussion

This study is the first to demonstrate that community-dwelling older adults with irregular mealtimes have higher serum GDF-15 levels, a potential biomarker of biological aging, and that approximately 15% of this association is explained by lower dietary quality (i.e., lower nutrient density). Individuals with mealtime irregularity had significantly lower intake of fish and seafood than those with regular mealtimes. In the LMG analysis examining the contribution of 17 food groups to nutrient density (NRF9.2), fish and seafood showed the second-largest contribution after vegetables. These findings indicate that older adults with irregular mealtimes have higher GDF-15 levels, and that part of this association may be attributable to poorer dietary quality, with lower intake of fish and seafood as one contributing factor.

Although the contribution of legumes and beverages to the NRF9.2 score was small, their intakes also differed between the irregular and regular mealtime groups. Legume intake was higher in the irregular group than in the regular group. In Japan, tofu and natto are commonly consumed and require no cooking, making them easy to include in daily meals. In the NRF9.2 calculation, higher scores are achieved when intakes of protein, dietary fiber, vitamins A, C, and D, calcium, iron, potassium, and magnesium meet recommended levels [29–31]. Although legumes are rich in protein, calcium, magnesium, and iron, fish and seafood generally provide more protein and iron at typical consumption levels, and legumes contain no vitamin D [28]. By contrast, fish are a major source of vitamin D [28]. Consequently, the contribution of legumes to the NRF9.2 score (2.4%, ranked 9th) was much lower than that of fish and seafood (14.5%, ranked 2nd). Beverage intake was lower in the irregular group than in the regular group. The beverage classification used in this study, based on the Japanese Standard Tables of Food Composition [28], includes green tea and coffee, which are known to have beneficial health effects. Although detailed consumption of these beverages could not be assessed in this study, they serve as sources of vitamin C, potassium, and magnesium [28]. Thus, these dietary characteristics of the irregular group likely contributed to their lower nutrient density.

GDF-15 is a cytokine belonging to the transforming growth factor-β superfamily and is strongly induced by cellular stresses, including mitochondrial dysfunction, oxidative stress, and inflammation [14]. Recently, GDF-15 has been increasingly recognized as an indicator of biological aging because of its close association with mitochondrial dysfunction [10–14]. Studies in older adults have shown that elevated GDF-15 levels are a strong predictor of mortality risk [34–36]. In our study, older adults with irregular mealtimes had significantly higher GDF-15 levels than those with regular mealtimes, despite being significantly younger in terms of chronological age. This association remained significant even after adjusting for stress-related factors known to influence GDF-15, including inflammatory factors such as smoking and chronic diseases. These findings suggest that irregular mealtimes are associated with the progression of biological aging. However, GDF-15 is a highly non-specific and pleiotropic biomarker influenced by a wide range of physiological and pathological processes [37–39]. Therefore, factors other than dietary quality may also mediate or confound the association between irregular mealtimes and GDF-15 levels. Indeed, dietary quality explained only approximately 15% of this association in our mediation analysis, suggesting that multiple pathways are likely involved. Given the cross-sectional design, the temporal direction of these relationships cannot be determined, and the observed associations should be interpreted with caution.

Many epidemiological studies have reported an association between dietary quality and health outcomes in older adults. In a large Canadian cohort, higher dietary quality was associated with better maintenance of cardiometabolic health [23]. A recent systematic review highlights the role of high-quality diets in promoting healthy aging [40], and another review indicates that adherence to healthy dietary patterns may slow the progression of biological aging [41]. The evidence from these previous studies strengthens our finding that dietary quality may be one pathway linking irregular mealtimes to accelerated biological aging.

In older adults, reduced physical activity lowers energy requirements, which can lead to nutrient deficiencies even when total energy intake appears adequate; therefore, consuming a nutrient-dense diet is particularly important [42]. Indeed, previous studies have shown that higher nutrient density is associated with a lower risk of all-cause mortality [43]. In the present study, low dietary nutrient density partially explained the positive association between irregular mealtimes and GDF-15 levels. These findings provide a basis for future studies to determine whether improving nutrient density can slow the progression of biological aging in older adults with irregular mealtimes. Advancing this line of research may ultimately enable the development of specific dietary support strategies for older individuals who have difficulty maintaining regular mealtimes, such as those whose eating schedules are disrupted by shift work, by clarifying *what* and *when* they should eat.

This study had some important limitations. First, the cross-sectional design represents a major limitation, as causal relationships cannot be established. Reverse causation, biological aging leading to mealtime irregularity, cannot be ruled out; for instance, factors related to underlying health status could affect GDF‑15 levels and the regularity of mealtimes, making it difficult to determine the direction of the association. Second, the study population consisted of older adults from a single geographic area and was relatively small. Participants who attended the in-person survey may have been healthier and more health-conscious than the general older population, which may increase the risk of selection bias and limit the generalizability of the findings. Consequently, the external validity of the results may be limited. Third, although the 3DR is considered the gold‑standard method, participants may alter their usual eating behavior while recording their intake, and the 3DR may not fully capture habitual dietary patterns. Although Food Frequency Questionnaires are designed to reflect habitual dietary intake, they rely heavily on memory, raising concerns about reliability in older populations. Therefore, the 3DR was adopted in this study. Fourth, although the analyses were guided by a priori hypotheses grounded in established biological mechanisms, they were conducted as a secondary analysis of existing data. Despite adjusting for major determinants of GDF-15 levels, such as kidney disease, cancer, and respiratory disease, residual confounding cannot be completely eliminated, as GDF-15 is influenced by a wide range of physiological and pathological conditions [37–39]. These may include factors not fully captured in our covariates, such as frailty or socioeconomic status. Furthermore, the possibility of chance findings cannot be entirely ruled out. Finally, mealtime irregularity in this study was defined based on a subjective questionnaire. Because no unified standard exists for assessing mealtime irregularity, the item used in this study has not been formally validated, and subjective judgments may vary across individuals. Consequently, some degree of misclassification cannot be ruled out.

Overall, these limitations highlight the need for future longitudinal studies in larger and more diverse populations that incorporate objective assessments of eating habits and dietary intake, and that consider broader determinants of GDF-15. In particular, future studies should use more objective approaches to capture meal timing patterns, including digital tools, given that subjective self-reports may introduce individual variability and potential misclassification. In addition, intervention studies are needed to determine whether improving dietary quality can slow the progression of biological aging in older adults with irregular mealtimes. Such research will be crucial to determine whether improving nutrient quality can effectively slow the progression of biological aging in older adults with mealtime irregularities.

## Conclusion

In this study, mealtime irregularity was associated with higher GDF-15 levels, a biomarker of biological aging, among community-dwelling older adults. This association was partially explained by poor dietary quality. Furthermore, our findings suggest that a lower fish intake contributes to the reduced dietary quality observed in individuals with irregular mealtimes. Our results provide valuable insights for designing future studies to determine whether improving dietary quality can help slow the progression of biological aging, even among those who cannot maintain regular mealtimes. This evidence may ultimately support the development of targeted dietary strategies for people with irregular mealtimes.

## Data Availability

The datasets analyzed in this study are not publicly available because the participants did not provide consent for data sharing.
